# The Role of PPARγ in Advanced Glycation End Products-Induced Inflammatory Response in Human Chondrocytes

**DOI:** 10.1371/journal.pone.0125776

**Published:** 2015-05-29

**Authors:** Chi Ma, Ying Zhang, Yu-qing Li, Cheng Chen, Wei Cai, Yue-lin Zeng

**Affiliations:** Department of Orthopedics, 163 Central Hospital of the People's Liberation Army, the Second Affiliated Hospital of Hunan Normal University, Changsha, Hunan, 410003, China; University of Kentucky Medical Center, UNITED STATES

## Abstract

**Objective:**

Advances made in the past ten years highlight the notion that peroxisome proliferator-activated receptors gamma (PPARγ) has protective properties in the pathophysiology of osteoarthritis (OA). The aim of this study was to define the roles of PPARγ in AGEs-induced inflammatory response in human chondrocytes.

**Methods:**

Primary human chondrocytes were stimulated with AGEs in the presence or absence of neutralizing antibody against RAGE (anti-RAGE), MAPK specific inhibitors and PPARγ agonist pioglitazone. The expression of IL-1, MMP-13, TNF-α, PPARγ, nuclear NF-κB p65 and cytosol IκBα was determined by western blotting and real-time PCR.

**Results:**

AGEs could enhance the expression of IL-1, TNF-α, and MMP-13, but the level of PPARγ was decreased in a time- and dose-dependent manner, which was inhibited by anti-RAGE, SB203580 (P38 MAPK specific inhibitor) and SP600125 (a selective inhibitor of JNK). PPARγ agonist pioglitazone could inhibit the effects of AGEs-induced inflammatory response and PPARγ down-regulation. In human chondrocytes, AGEs could induce cytosol IκBα degradation and increase the level of nuclear NF-κB p65, which was inhibited by PPARγ agonist pioglitazone.

**Conclusions:**

In primary human chondrocytes, AGEs could down-regulate PPARγ expression and increase the inflammatory mediators, which could be reversed by PPARγ agonist pioglitazone. Activation of RAGE by AGEs triggers a cascade of downstream signaling, including MAPK JNK/ p38, PPARγ and NF-κB. Taken together, PPARγ could be a potential target for pharmacologic intervention in the treatment of OA.

## Introduction

Accumulating evidence have shown that osteoarthritis (OA) is a classic age-related disease [1,2]. A prominent feature of aging is the accumulation of advanced glycation end products (AGEs) resulting from spontaneous reaction of reducing sugars with proteins or non-enzymatic glycation[3,4]. Numerous studies have suggested that AGES and their receptor (RAGE) axis are implicated in the pathogenesis and progression of OA [5,6]. However, the details of the mechanisms involved remain largely unknown.

Peroxisome proliferator-activated receptors gamma (PPARγ) is a member of the ligand activated nuclear hormone receptor superfamily[7]. Although PPARγ exhibits the function of regulating fatty acid uptake, insulin sensitivity and glucose homeostasis, whether it plays a crucial role in AGEs induced chondrocyte damage has not been clearly determined. Accumulating data have indicated that the expression of PPARγ is decreased in OA chondrocytes [5,8] and synovial fibroblasts [9]. Pioglitazone, one of PPARγ agonists, has been confirmed that it is in a position to inhibit the progression of guinea pig OA [8]. Taken together, we put forward the hypothesis for the first time that PPARγ down-regulation in chondrocytes might be responsible for AGEs-induced production of TNF-α and MMP-13. Our previous study has indicated that the expression of PPARγ was decreased when rabbit chondrocytes were stimulated with AGEs [10]. The current study was designed to define the roles of PPARγ in AGEs-induced inflammatory response in human chondrocytes and investigate whether PPARγ agonists pioglitazone could inhibit the effects of AGEs on primary human chondrocytes.

## Methods and Materials

### Ethics Statement

The samples of articular cartilage collection were approved by the Research Ethics Committee of the Second Affiliated Hospital of Hunan Normal University, China. A written informed consent was also obtained from the patients.

### Reagents and Antibodies

MMP-13 antibody was purchased from Santa Cruz Biotechnology (Santa Cruz, CA, USA). Rabbit monoclonal antibodies specific for IL-1β, NF-κB p65, PPARγ, TNF-α, IκBα, β-actin and RAGE were purchased from Cell signaling Technology (Danvers, MA, USA). Rabbit polyclonal antibody specific for IL-1α were purchased from Abcam (San Francisco, CA, USA). SB203580, SP600125, PD98059, BAY-11-7082 and Pioglitazone were purchased from Cayman Chemical Company (U.S.A). Advanced Glycation End Product (AGE)-BSA was purchased from BioVision, Inc (USA). Penicillin/streptomycin solution, fetal bovine serum (FBS), low-glucose Dulbecco’s modified Eagle’s medium (DMEM), type II collagenase, and trypsin were purchased from Invitrogen (Carlsbad, CA, USA). All other chemicals were obtained from Sigma-Aldrich (St. Louis, MO, Germany) unless indicated otherwise.

### Isolation and Culture Chondrocyte from Human Articular Cartilage

Human articular cartilage specimens were obtained under aseptic conditions from 6 patients aged 28-44 years (mean age, 31.2±2.91 years) who were generally healthy undergoing knee amputations for sever trauma. Cartilage was cut into 1 cubic millimeter finely, and chondrocytes were isolated by sequential enzymatic digestion at 37°C with 0.25% trypsin for 40 minutes and 2mg/ml type II collagenase for five hours in low-glucose DMEM. After filtration, the chondrocytes were grown in complete low-glucose DMEM (supplemented with 15% FBS, 100 U/ml penicillin and 100 μg/ml streptomycin). At 80-90% confluence, the cells were passaged once and seeded at high density. All experiments were conducted using chondrocytes within 1-4 passages. In experiments, the chondrocytes were seeded at 2×10^5^ cells per well in 6-well plates and treated with 1-100 μg/ml AGEs for various time intervals in the presence or absence of antibodies for RAGE (anti-RAGE) and pharmacological inhibitors for NF-κB or MAPKs.

### Cell Lysate Preparation

According to the protocol of the kit (Viagene Biotech, Ningbo, China), cytoplasmic and nuclear extracts were extracted and isolated. Briefly, the chondrocytes were washed three times with cold PBS and lysed with cytoplasmic protein extraction reagent Ⅰ and Ⅱ. When the lysate was centrifuged, the supernatant was immediately frozen at -80°C for later analysis of cytoplasmic proteins. The precipitation were resuspended in nuclear protein extracion agent and voretexed forcibly on ice every 3 minutes for a total 30 minutes. After being centrifuged for 10 min at 4°C, the supernatants were collected as nuclear extracs protein. For isolation of total cell extracts, the cells were washed twice with cold PBS and then resuspended in lysis buffer containg 1mM phenylmethanesulfonyl fluoride (PMSF) on ice for 30 minutes. The lysate was centrifuged at 12,000×g for 15 min at 4°C, and the supernatant was collected. Protein concentration was quantified by BCA method. All samples were stored at − 80°C until further analysis.

### Western Blotting and Densitometric Analysis

Equal protein were run on a 10% SDS gel and transferred onto an immobilon-P (PVDF) membrane (Millipore). The blots were blocked with 5% nonfat milk in TBS at 4°C overnight. After being washed three times with TBS-T, the blots were probed with primary antibodies against MMP-13 (1:500), IL-1α (1:2000), IL-1β (1:1000), NF-κB p65 (1:1000), PPARγ (1:1000), TNF-α (1:1000), IKBα (1:1000) or β-actin (1:1000) at 4°C overnight. After being washed, the blots were subsequently incubated with the secondary goat anti-rabbit antibodies conjugated with horseradish peroxidase (1:1000) for 50 minutes at room temperature. The blots were visualized by enhanced chemiluminescence using the C-DiGit Blot Scanner (LI-COR Biosciences). Densitometric analysis of the scanned bands was performed using Image J (MD, USA) according to the manufacturer’s instructions.

### Total RNA Extraction and Real-time Fluorescent Quantitative PCR

Total RNA was extracted from chondrocytes using TRIzol (Invitrogen, USA) according to the protocol of the kit and dissolved in nuclease-free water. The total RNA was quantified by a spectrophotometer with ultraviolet light absorbance at 260 nm. The ration of optical density at wavelengths of 260 nm and 280 nm was used to assess the purity of the RNA. 1μg total RNA was reverse transcribed to synthesize cDNA according to the reverse transcription kit (Promega, USA).

cDNA obtained was amplified with real-time PCR on Bio-Rad CFX Connect real-time PCR detection system (Bio-Rad Laboratories, Inc) using QuantiFast SYBR Green PCR Kit (QIAGEN). Real-time PCR was performed using the specific primers as shown in Table 1. The real-time PCR reaction system is as follows: 5μl of Master Mix, 0.8μl of upstream primer, 0.8μl of downstream primer and 3.4μl Mili Q water. Reaction conditions were 95°C 5 minutes, 95°C 10 seconds, 60°C 30 seconds, 40cycles. The relative mRNA expression abundance was calculated by the formula *Y* = 2^-△△Ct^_._

**Table 1:** Primers for real-time fluorescent quantitative PCR.

Genes	Forward primer sequences (5’→3’)	Reverse primer sequences (5’→3’)
*MMP-13*	GGAAACCAGGTCTGGAGATATGA	TGGAATTTGCTGGCATGACG
*TNF-α*	GTAGCCCATGTTGTAGCAAACC	CTGATGGTGTGGGTGAGGAG
*IL-1α*	CAACCAGTGCTGCTGAAGGA	AGCACACCCAGTAGTCTTGC
*IL-1β*	AGGATATGGAGCAACAAGTGGT	AACACGCAGGACAGGTACAG
*PPARγ*	TTATTCTCAGTGGAGACCGCC	TGAGGACTCAGGGTGGTTCA
*β-actin*	TCATGAAGTGTGACGTGGACATC	CAGGAGGAGCAATGATCTTGATCT

### Statistical Analysis

The data are expressed as mean ± SD. The significance of differences was evaluated by using one-way ANOVA followed by Student’s *t*-test. The level of significant was set at *p<0*.*05*.

## Results

### Induction of IL-1α, IL-1β, TNF-α and MMP-13 Expression by AGEs in Primary Human Chondrocytes

**Fig 1:**
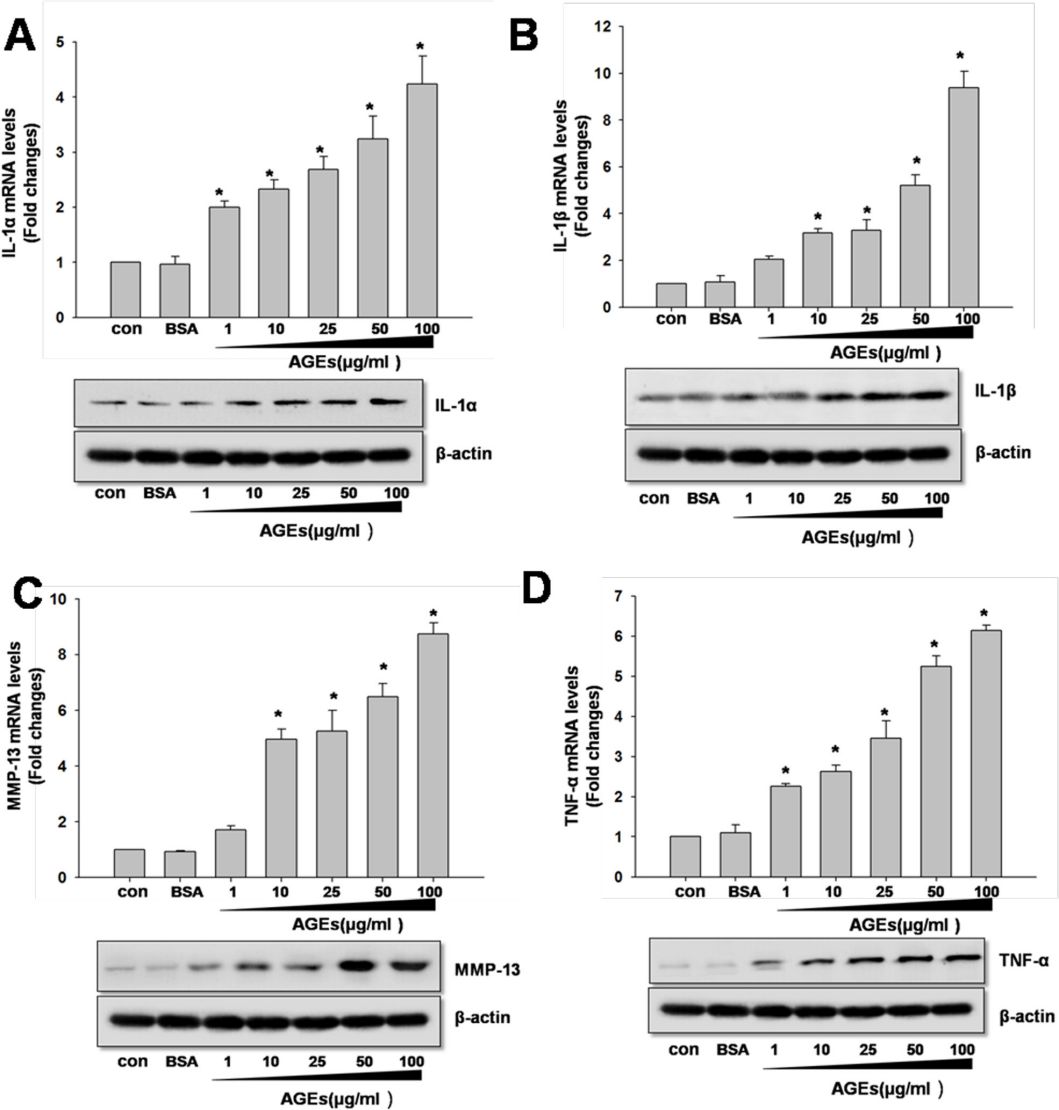
Induction of IL-1α, IL-1β, TNF-α and MMP-13 expression by AGE-BSA in primary human chondrocytes. Primary human chondrocytes were incubated with AGEs (1 to 100 μg/ml). The expression of IL-1α, IL-1β, TNF-α and MMP-13 was quantified by real-time PCR and western blotting using β-actin as an internal control. Densitometric analysis for IL-1α, IL-1β, TNF-α and MMP-13 levels corrected to β-actin is shown. All data are expressed as means ± SD and are representative of three independent experiments. *: *p< 0*.*05* versus control.

The chondrocytes were treated with AGE-BSA (1-100 μg/ml) for 0-24 hours. Our results showed that AGE-BSA could significantly up-regulate the expression of IL-1α, IL-1β, TNF-α and MMP-13 in a dose- and time-dependent manner (Fig 1). To note, the maximum effect was found to at 100 μg/ml AGEs. Therefore, subsequent experiments were performed with 100 μg/ml AGEs.

### Necessity of PPARγ for AGEs-induced Inflammatory Responses in Human Primary Chondrocytes

**Fig 2:**
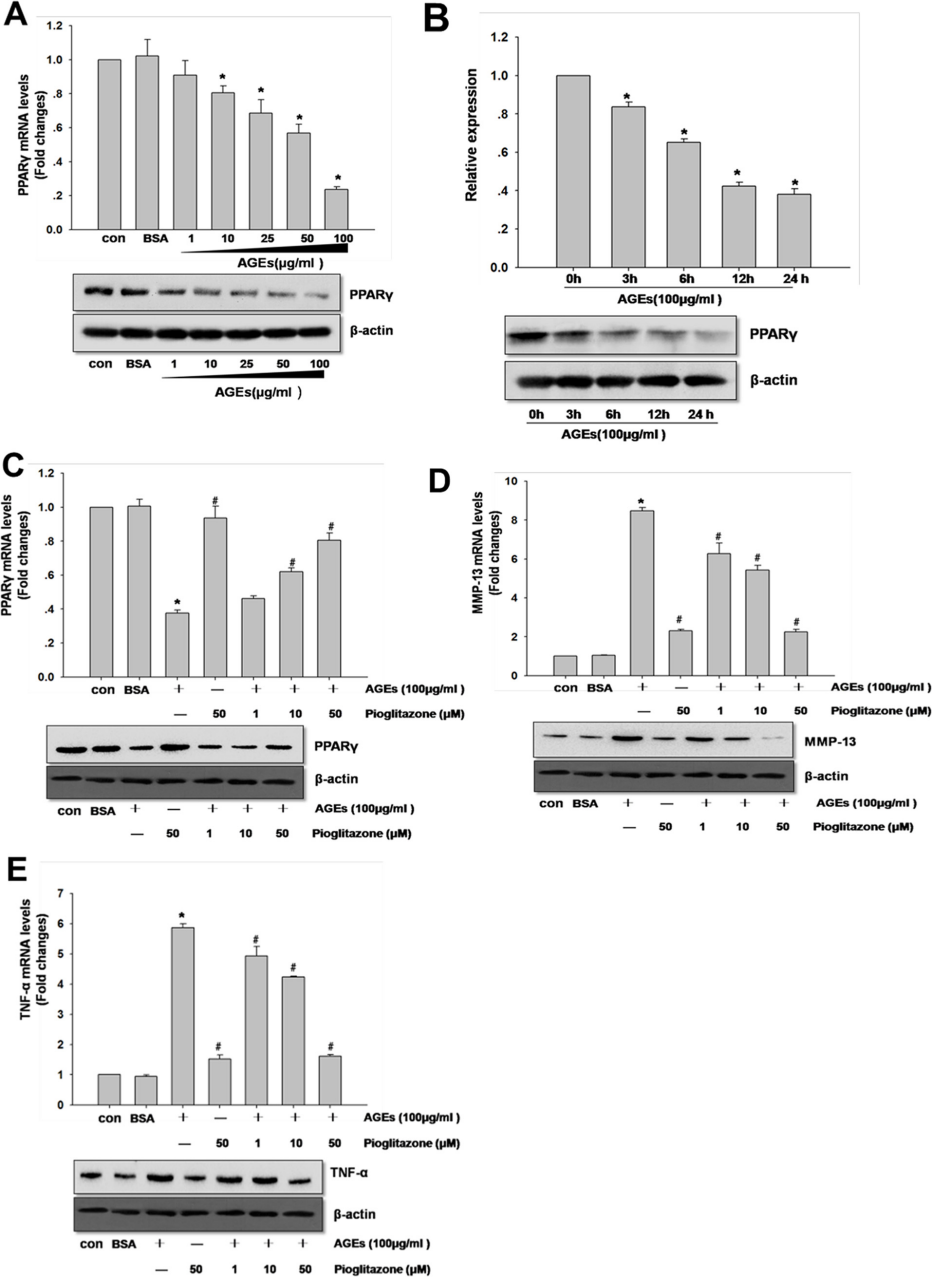
Necessity of PPARγ for AGEs-induced inflammatory responses in human primary chondrocytes. In A and B, chondrocytes were incubated with AGEs (1 to 100 μg/ml) for indicated time intervals (0-24 hours). In C, D and E, chondrocytes were pretreated with pioglitazone for 2 hours AGEs (100 μg/ml) stimulation. The expression of PPARγ, TNF-α and MMP-13 was quantified by real-time PCR and western blotting using β-actin as an internal control. In B, densitometric analysis for PPARγ levels corrected to β-actin is shown. All data are expressed as means ± SD and are representative of three independent experiments. *: *p< 0*.*05* versus control, #: *p< 0*.*05* versus AGEs treatment.

In human chondrocytes, AGEs also could down-regulate PPARγ expression in a time- and dose-dependent manner (Fig 2A and 2B). In order to investigate whether AGEs-induced inflammatory responses in primary human chondrocytes was mediated by PPARγ, the cells were pre-incubated with PPARγ agonist pioglitazone for 12 hours prior to AGEs stimulation. Our results showed that pioglitazone could reverse the effect of AGEs-induced PPARγ, TNF-α and MMP-13 expression in a dose-dependent manner (Fig 2C, 2D and 2E).

### Effects of Anti-RAGE and MAPKs inhibitor on AGEs-induced Down-regulation of PPARγ

**Fig 3:**
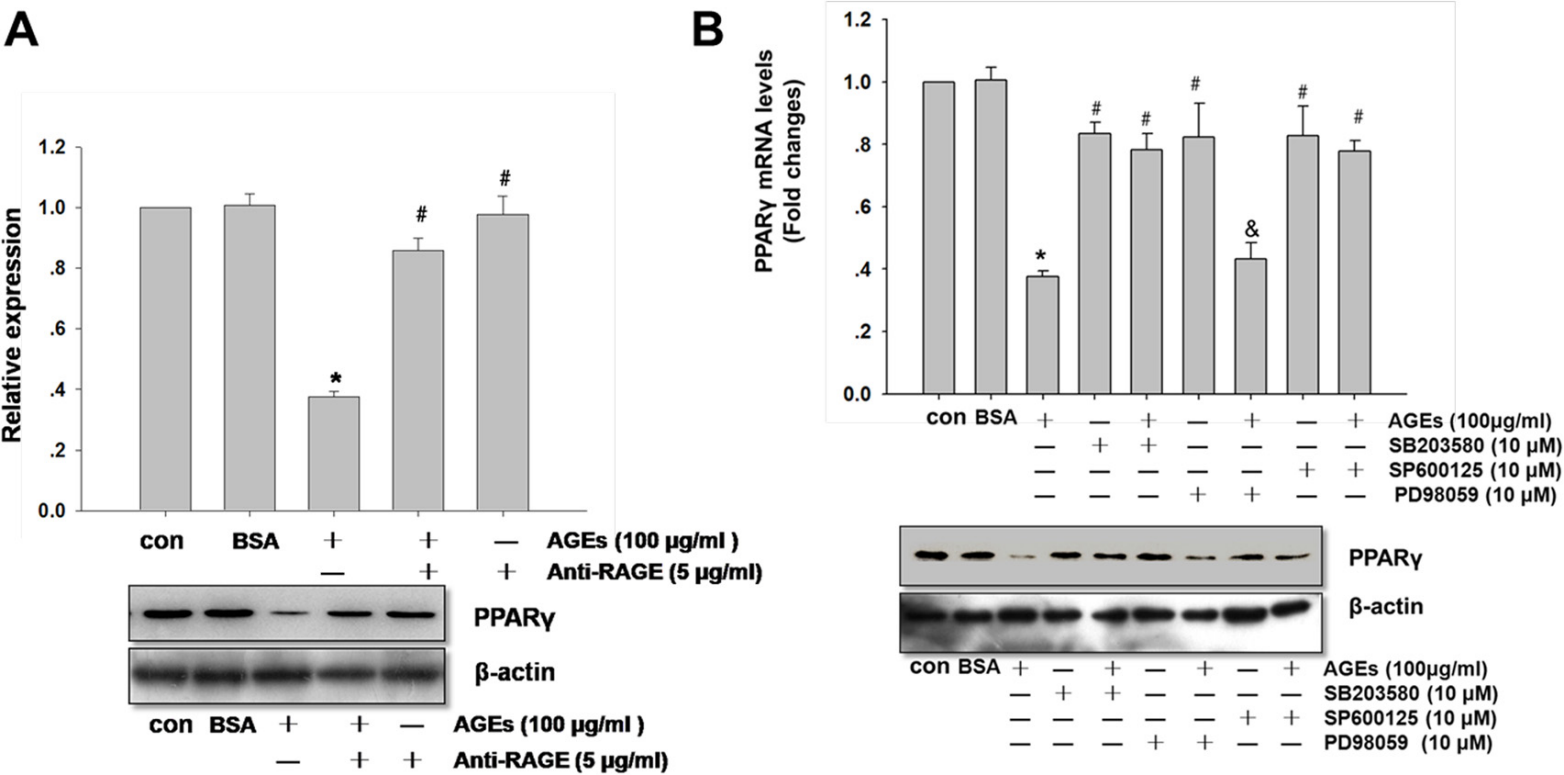
Effects of anti-RAGE and MAPKs inhibitor on AGEs-induced down-regulation of PPARγ in primary human chondrocytes. In A, chondrocytes were pretreated with anti-RAGE (5 μg/ml) for 12 hours before AGEs (100 μg/ml) stimulation. In B, chondrocytes were pretreated with SB203580, SP600125, PD98059 (10 μM) for 30 minutes prior to AGEs (100 μg/ml) stimulation. The expression of PPARγ was quantified by real-time PCR and western blotting using β-actin as an internal control. In A, densitometric analysis for PPARγ levels corrected to β-actin is shown. All data are expressed as means ± SD and are representative of three independent experiments. *: *p< 0*.*05* versus control, #: *p< 0*.*05* versus AGEs treatment, &: *p> 0*.*05* versus AGEs treatment.

To gain further insights into whether RAGE and MAPK pathway were involved in AGEs-induced PPARγ down-regulation. Chondrocytes were pretreated with anti-RAGE (5 μg/ml), SB203580 (P38 MAPK specific inhibitor), SP600125 (a selective inhibitor of JNK) and PD98059 (a selective inhibitor of ERK) prior to AGEs (100 μg/ml) stimulation. In Fig 3A, anti-RAGE could significantly inhibit the effect of AGEs on the down-regulation of PPARγ. In Fig 3B, SB203580 or SP600125 could significantly revert the effect of AGEs-induced down-regulation of PPARγ. However, PD98059 had no significant effect on AGEs-induced down-regulation of PPARγ.

### The Effect of Pioglitazone on AGEs-induced Activation of NF-κB in Primary Human Chondrocytes

**Fig 4:**
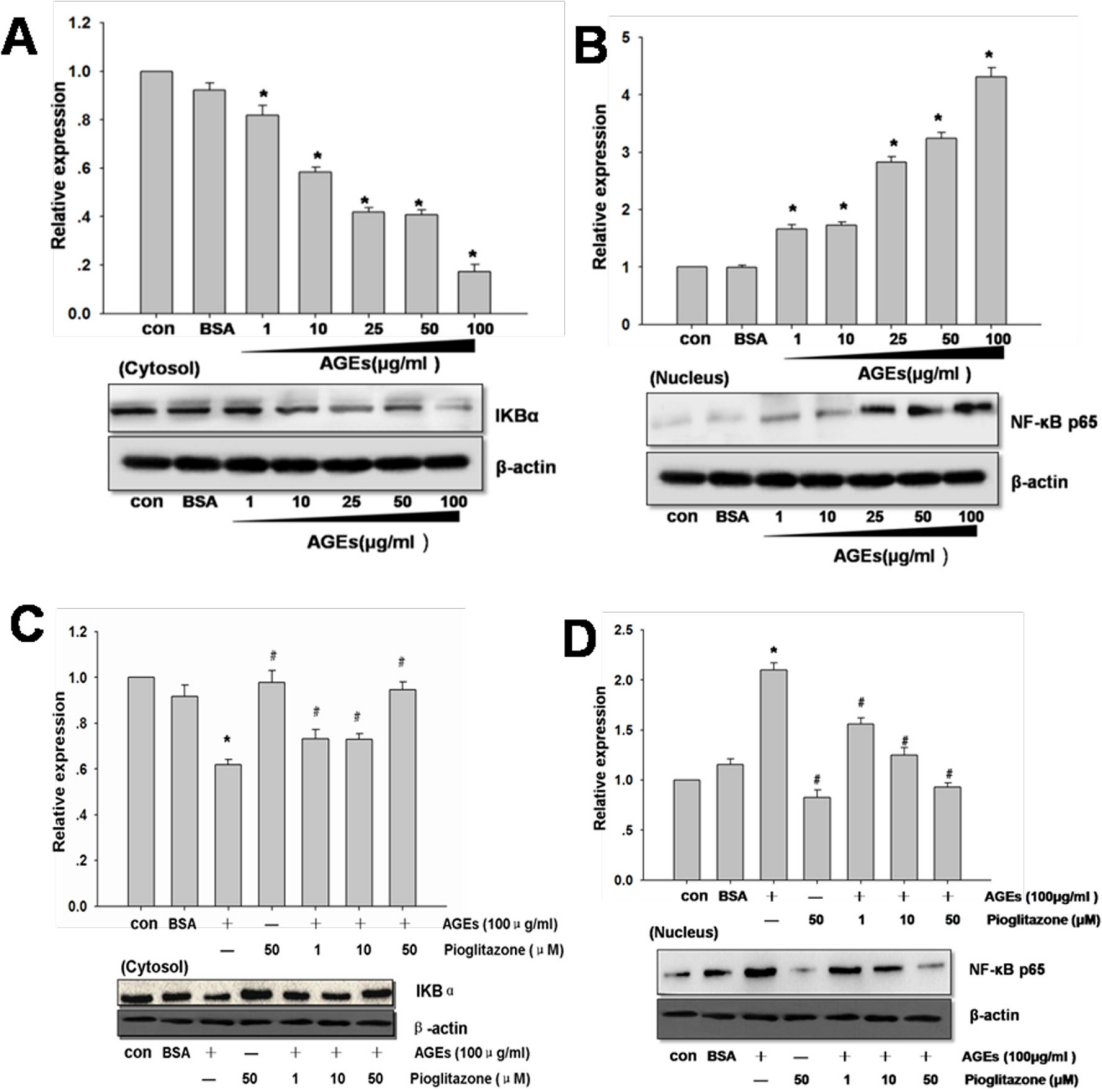
The effect of pioglitazone on AGEs-induced activation of NF-κB in primary human chondrocytes. Primary human chondrocytes were incubated with AGEs (1 to 100 μg/ml) for the indicated time intervals. The levels of cytosol IκBα (A) and nuclear NF-κB p65 (B) were determined by western blotting. In C and D, the chondrocytes were pretreated with pioglitazone (1-50 μM) for 2 hours and then incubated with AGEs (100 μg/ml) for 24 hours. The level of cytosol IκBα (C) and nuclear NF-κB p65 (D) were determined by western blotting. Densitometric analysis for NF-κB p65 and IκBα levels corrected to β-actin is shown. All data are expressed as means ± SD and are representative of three independent experiments. *: *p< 0*.*05* versus control.

Firstly, the chondrocytes were treated with 1-100 μg/ml AGEs for various time intervals. In Fig 4A and 4B, chondrocytes stimulated with AGEs could induce cytosol IκBα degradation and increase the level of nuclear NF-κB p65. These results indicated that AGEs could increase the activity of NF-κB. To further investigate the relationship between NF-κB pathway and PPARγ, we next used pioglitazone to pretreat human chondrocytes prior to stimulation with AGEs. As shown in Fig 4C and 4D, pioglitazone could dose-dependently inhibit AGEs-induced cytosol IκBα degradation and the level of nuclear NF-κB p65.

## Discussion

OA, the most prevalent musculoskeletal disorder, is a progressive degenerative joint disease leading to significant functional impairment and disability in older adults[1,2]. It is characterized by inhibiting the production of anabolic factors and releasing more catabolic factors, which results in further damage to the cartilage, and leads to the release of matrix components and induce the inflammatory response[11]. OA is a classic age-related disease and a prominent feature of aging is the accumulation of AGEs. In vivo, the generation of AGEs is an inevitable process and the concentration of AGEs in OA joint tissue up to 56.6±28.7nmol/l, which is the 5-fold of normal tissue[12]. So the concentration of AGEs used in the experiment is according to the previous study. In addition, we used commercially available AGE-BSA, a complex that includes CML, pentosidine, and other AGE, instead of a specific AGE, usually pentosidine or N3-carboxymethyllysine (CML).

The peroxisome proliferator-activated receptors (PPAR) belong to the nuclear hormone receptor superfamily and have a diverse role in a wide ranged of tissues, including the regulation of glucid and lipid metabolism, inflammation, and diabetes [13,14]. Advances made in the past ten years highlight the notion that PPARγ has protective properties in the pathophysiology of OA [5,8,10,15,16,17,18]. Nebbaki et al have reported that in Hartley guinea pig and the anterior cruciate ligament transaction dog models, the level of PPARγ was decreased during the progression of the disease and it was correlated negatively with the severity of OA[15]. Cartilage-specific PPARγ knockout mice exhibit the spontaneous osteoarthritis phenotype, which further provide evidence that PPARγ is a critical regulator of cartilage health [16]. Studies have demonstrated that PPARγ agonist pioglitazone could reduce the severity of OA in animal models [8,17]. In addition, inflammatory and catabolic responses in rheumatoid synovial fibroblasts and monocytes could be inhibited by PPARγ activation[10,19]. Simultaneously, our data have shown that PPARγ agonist pioglitazone could decrease the level of IL-1α, IL-1β, MMP-13 and TNF-αin human chondrocytes.

There is increasing evidence that PPARγ was involved in AGEs-related disease, including diabetes and diabetes-related complications[20,21,22,23,24,25], cardiovascular disease[26], non-alcoholic fatty liver disease (NAFLD)[27], cognitive dysfunction and dementia[28], and OA[5,10]. Our previous study have suggested that AGEs could time- and dose-dependently decrease the expression of PPARγ in rabbit chondrocytes[10]. In the present study, we further demonstrated that the level of PPARγ was decreased in primary human chondrocytes, whereas the expression of IL-1, MMP-13 and TNF-α was increased, and this effect could be inhibited by pioglitazone. Collectively, theses data indicated that PPARγ plays an important role in AGEs-induced human chondrocytes OA model.

To further investigate the intracellular signaling mechanism for the effects of AGEs on the expression of PPARγ, the neutralizing antibody against RAGE (anti-RAGE) was pre-incubated with human chondrocytes prior to AGEs treatment. Anti-RAGE could significantly inhibit the effect of AGEs on the down-regulation of PPARγ, which provides strong evidence for the involvement of RAGE in AGEs mediated PPARγ down-regulation in human chondrocytes. The interaction of AGEs with RAGE has been shown to activate multiple cellular signaling cascades in chondrocytes and other cells, including the MAPK pathway through increasing the phosphorylation level of MAPK[29,30,31,32]. Moreover, it has been demonstrated that the activation of MAPK signaling pathways could increase the level of the mediators of cartilage catabolism such as MMPs and TNF-α[6,33,34]. SB203580, PD98059, SP600125 were utilized to prestimulate the chondrocytes for 30 minutes before the chondrocytes were stimulated with AGEs. It is noteworthy that SB203580 or SP600125 could significantly up-regulate PPARγ expression in AGEs-stimulated human chondrocytes, whereas PD98059 had no significant effect on AGEs-induced down-regulation of PPARγ. Our current data have confirmed and extended our previous study in rabbit chondrocytes that MAPK JNK and p38, but not ERK, were involved in the mechanism of AGEs-induced down-regulation of PPARγ in human chondrocytes. Its potential mechanisms may be that the activation MAPK JNK and p38 could increase the level of phosphorylation of PPARγ which leads to the decrease of the PPARγ transcriptional activity and helps translocation of PPARγ to the cytoplasm [35,36].

To further investigate the necessity of NF-κB for AGEs-induced PPARγ down-regulation, the chondrocytes were incubated with AGEs. AGEs could increase the NF-κB activity in a time- and dose-dependent manner, and induce cytosol IκBα degradation and increase the level of nuclear NF-κB p65. Interestingly, PPARγ agonist pioglitazone could reverse this effect. Our results are consistent with the findings of Mahali and his colleagues [35], whose results showed that AGEs-dependent increase of NF-κB and decrease in PPARγ DNA binding activities are inversely correlated. These findings indicated that NF-κB pathway is involved in PPARγ down-regulation in AGEs-stimulated human chondrocytes.

**Fig 5:**
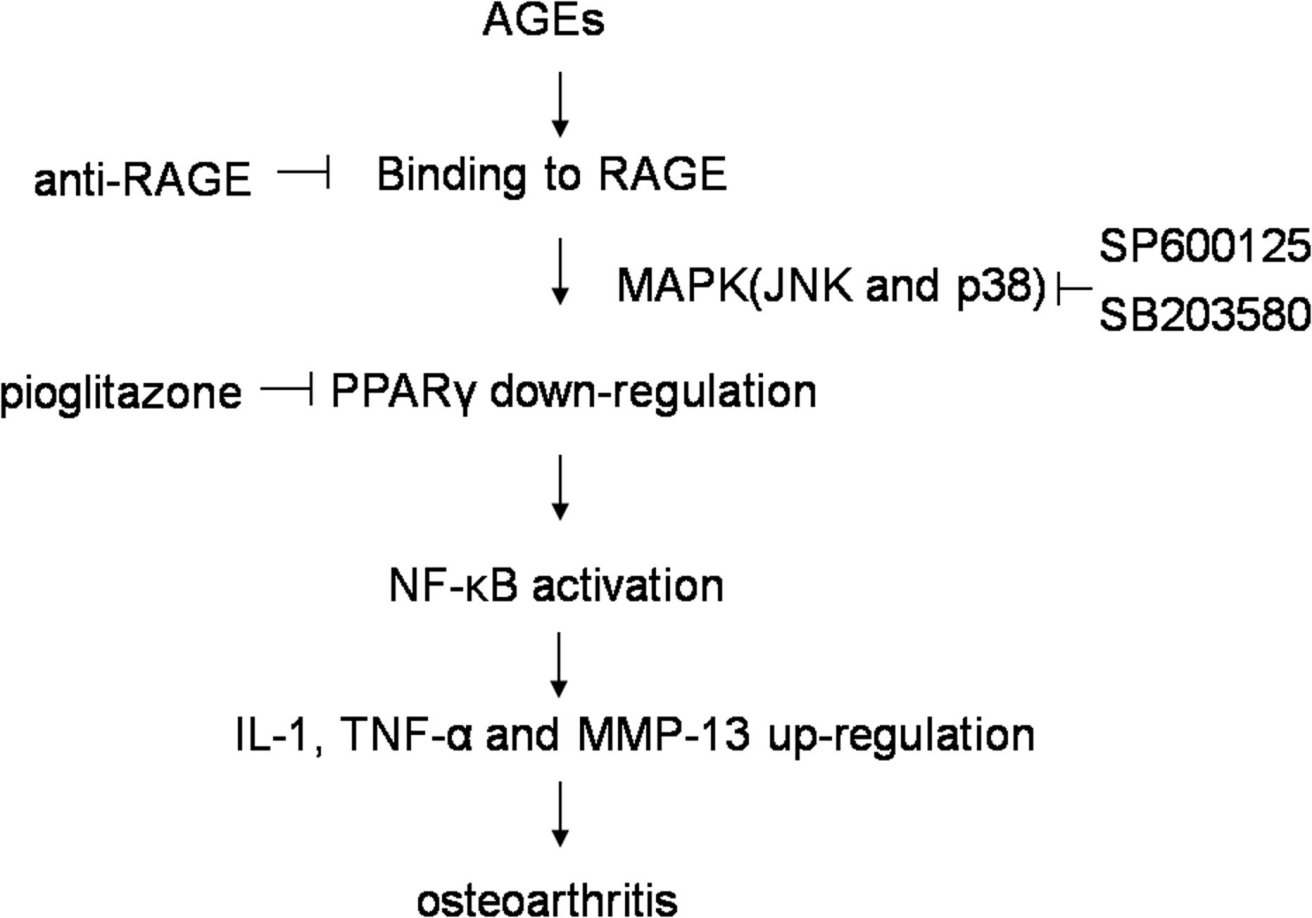
Proposed scheme of AGEs-induced IL-1, TNF-α and MMP-13 expression mediated by down-regulating PPARγ through MAPK and NF-κB signaling pathway.

In conclusion, our study demonstrates that AGEs down-regulate PPARγ via RAGE in primary human chondrocytes. Activation of RAGE by AGEs triggers a cascade of downstream signaling, including the activation of MAPK JNK/ p38, the down-regulation of PPARγ and the activation NF-κB (Fig 5). Taken together, PPARγ could be a potential target for pharmacologic intervention in the treatment of OA.
